# Supplementary material for: Anti-Acanthamoeba Activity of Brominated Sesquiterpenes from Laurencia johnstonii
Source: Mar Drugs. 2018 Nov 11;16(11):443. doi: 10.3390/md16110443 (PMC6266398; doi:10.3390/md16110443)

Supporting Information

Anti-*Acanthamoeba* activity of brominated sesquiterpenes from *Laurencia johnstonii*

Sara García-Davis[1,2], Ines Sifaoui[3], María Reyes-Batlle[3], Ezequiel Viveros-Valdez[2], José E. Piñero[3], Jacob Lorenzo-Morales[3], José J. Fernández[1,4*] and Ana R. Díaz-Marrero[1*]

[1] Instituto Universitario de Bio-Orgánica Antonio González (IUBO AG), Centro de Investigaciones Biomédicas de Canarias (CIBICAN), Universidad de La Laguna (ULL), Avda. Astrofísico F. Sánchez, 2, 38206 La Laguna, Tenerife, España; sara.garciadv@uanl.edu.mx (S.G.D.); adiazmar@ull.edu.es (A.R.D.M); jjfercas@ull.edu.es (J.J.F.)

[2] Universidad Autónoma de Nuevo León (UANL), Facultad de Ciencias Biológicas. Av. Pedro de Alba s/n, 66450 San Nicolás de los Garza, Nuevo León, México; jose.viverosvld@uanl.edu.mx (E.V.V.)

[3] Instituto Universitario de Enfermedades Tropicales y Salud Pública de Islas Canarias, Universidad de La Laguna. Av. Astrofísico Francisco Sánchez s/n, 38206 La Laguna, Tenerife, España.; ines.sifaoui@hotmail.com (I.S.); jmlorenz@ull.edu.es (J.L.M.)

[4] Departamento de Química Orgánica, Universidad de La Laguna (ULL), Avda. Astrofísico F. Sánchez, 2, 38206 La Laguna, Tenerife, España

TABLE OF CONTENTS

	Page
**Scheme S1.** Isolation process of sesquiterpenes **1**-**5** from *Laurencia johnstonii*	**3**
**Figure S1.** ^{1}H-NMR spectrum for laurinterol (**1**) (500 MHz, $CDCl_3$)	**4**
**Figure S2.** ^{1}H-NMR spectrum for isolaurinterol (**2**) (500 MHz, $CDCl_3$)	**5**
**Figure S3.** ^{1}H-NMR spectrum for aplysin (**3**) (500 MHz, $CDCl_3$)	**6**
**Figure S4.** ^{1}H-NMR spectrum for α-bromocuparane (**4**) (500 MHz, $CDCl_3$)	**7**
**Figure S5.** ^{1}H-NMR spectrum for α-isobromocuparane (**5**) (500 MHz, $CDCl_3$)	**8**
**Figure S6**. ^{1}H-NMR spectrum of 8-bromoaplysin (**6**) (600 MHz, $CDCl_3$)	**9**
**Figure S7.** COSY spectrum of 8-bromoaplysin (**6**) (600 MHz, $CDCl_3$)	**10**
**Figure S8.** HSQC-ed spectrum of 8-bromoaplysin (**6**) (600 MHz, $CDCl_3$)	**11**
**Figure S9.** HMBC spectrum of 8-bromoaplysin (**6**) (600 MHz, $CDCl_3$)	**12**
**Figure S10.** ^{13}C NMR spectrum of 8-bromoaplysin (**6**) (150 MHz, $CDCl_3$)	**13**
**Figure S11.** HREIMS spectrum of 8-bromoaplysin (**6**)	**14**
**Figure S12.** ^{1}H NMR spectrum of 3α-bromojohnstane (**7**) (600 MHz, $CDCl_3$)	**15**
**Figure S13.** COSY spectrum of 3α-bromojohnstane (**7**) (600 MHz, $CDCl_3$)	**16**
**Figure S14.** HSQC-ed spectrum of 3α-bromojohnstane (**7**) (600 MHz, $CDCl_3$)	**17**
**Figure S15.** HMBC spectrum of 3α-bromojohnstane (**7**) (600 MHz, $CDCl_3$)	**18**
**Figure S16.** ^{13}C NMR spectrum of 3α-bromojohnstane (**7**) (150 MHz, $CDCl_3$)	**19**
**Figure S17.** 1D-NOE experiments of 3α-bromojohnstane (**7**) (600 MHz, $CDCl_3$)	**20**
**Figure S18.** HREIMS spectrum of 3α-bromojohnstane (**7**)	**21**
**Figure S19.** ^{1}H NMR spectrum of 8,10-dibromoisoaplysin (**8**) (500 MHz, $CDCl_3$)	**22**
**Figure S20.** ^{1}H NMR spectrum of 8,10-dibromoaplysinol (**9**) (500 MHz, $CDCl_3$)	**23**

Scheme S1. Isolation process of sesquiterpenes **1**-**5** from *Laurencia johnstonii*

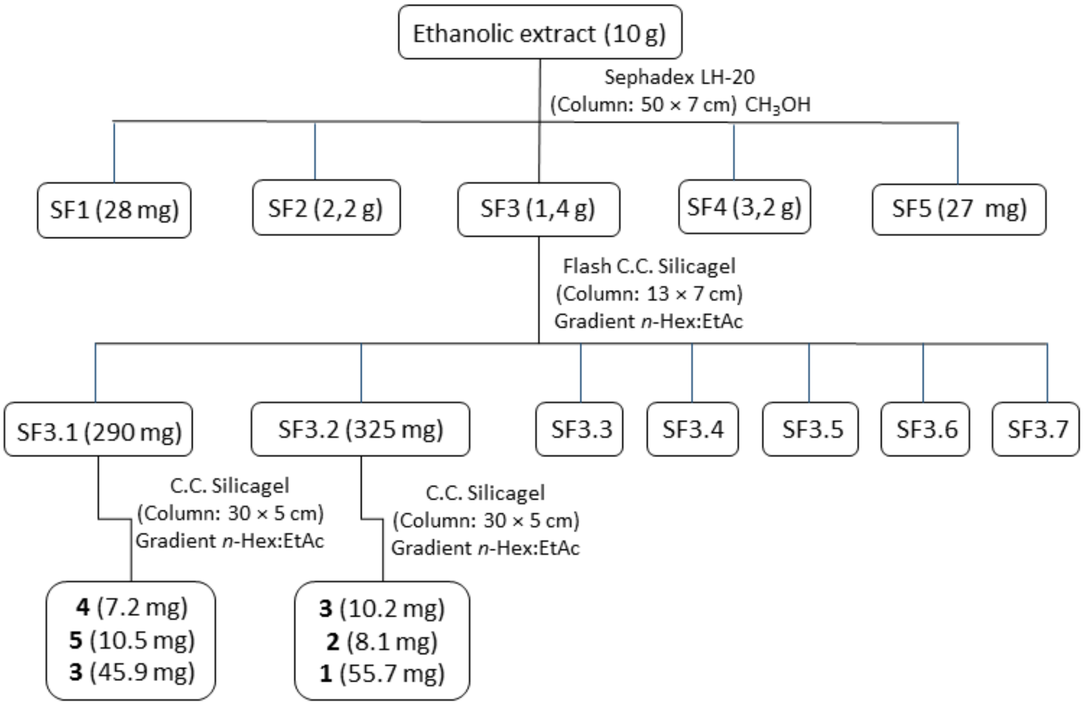

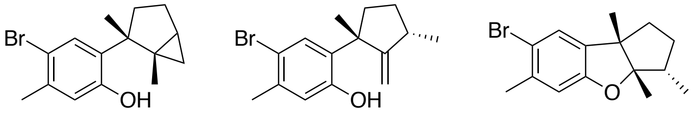

laurinterol (**1**) isolaurinterol (**2**) aplysin (**3**)

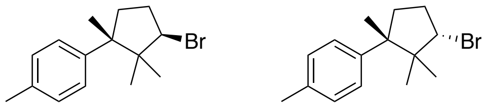

α-bromocuparane (**4**) α-isobromocuparane (**5**)

Figure S1. ^{1}H-NMR spectrum for laurinterol (**1**) (500 MHz, $CDCl_3$).

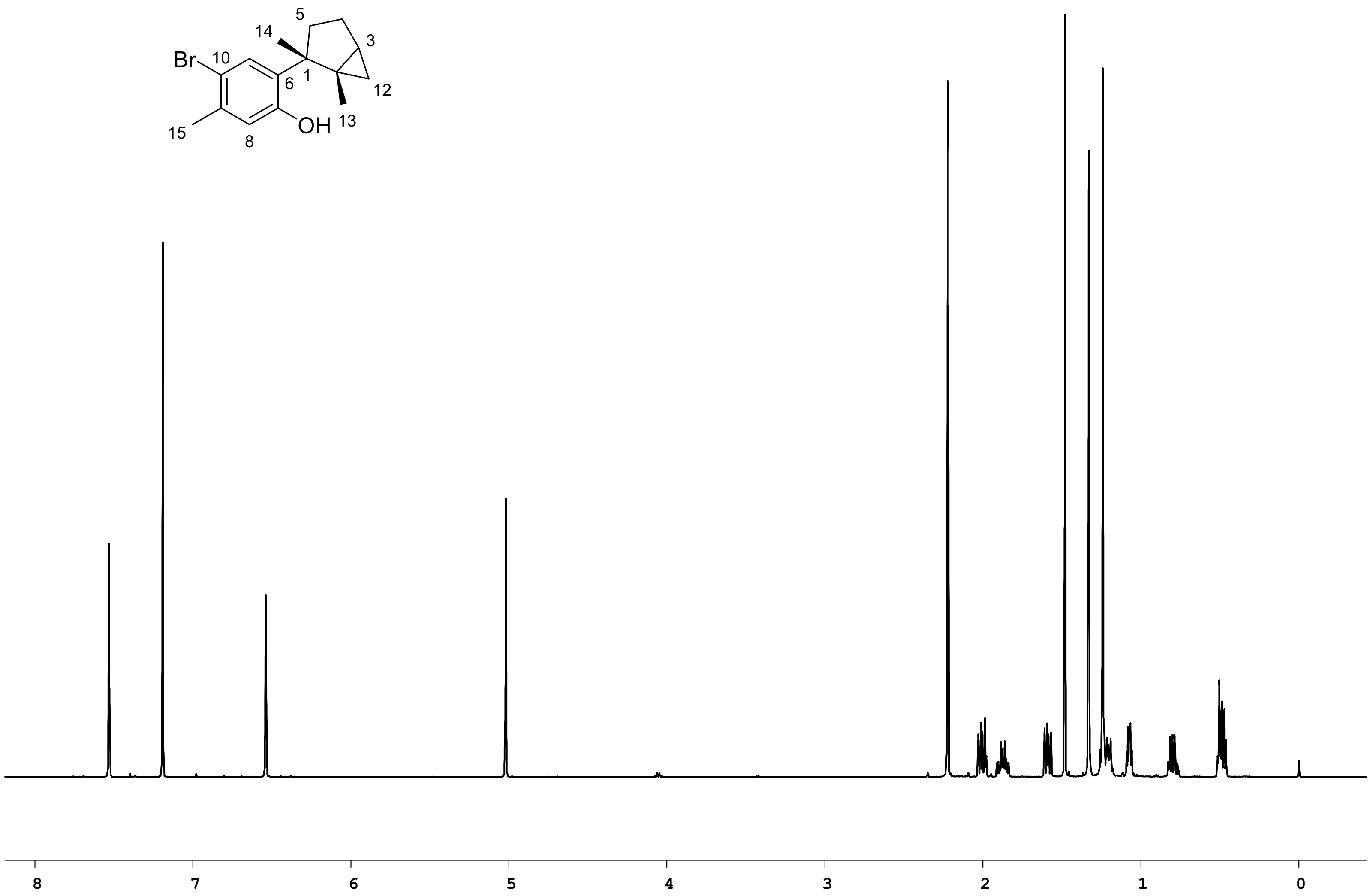

Figure S2. ^{1}H-NMR spectrum for isolaurinterol (**2**) (500 MHz, $CDCl_3$).

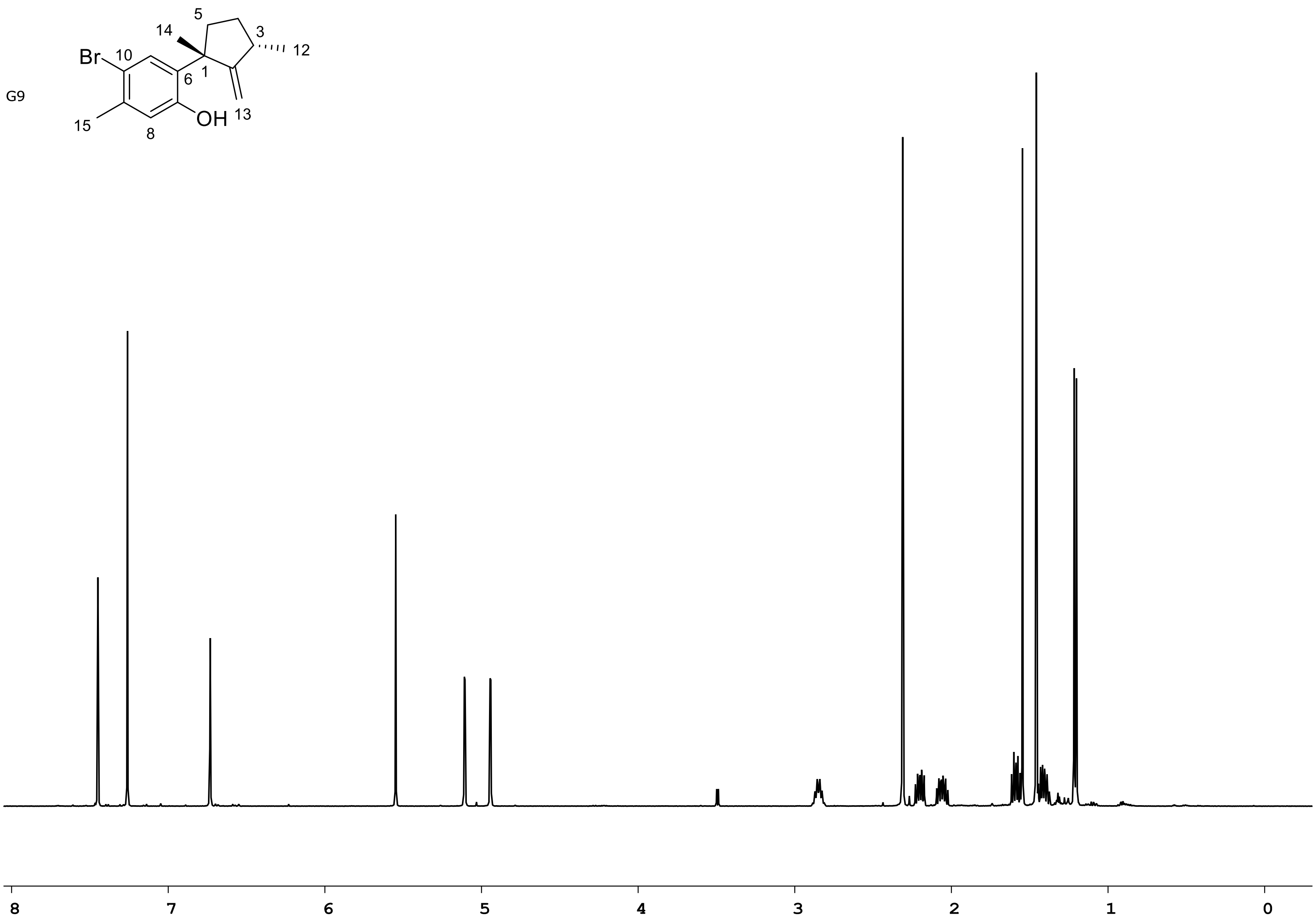

Figure S3. ^{1}H-NMR spectrum for aplysin (**3**) (500 MHz, $CDCl_3$).

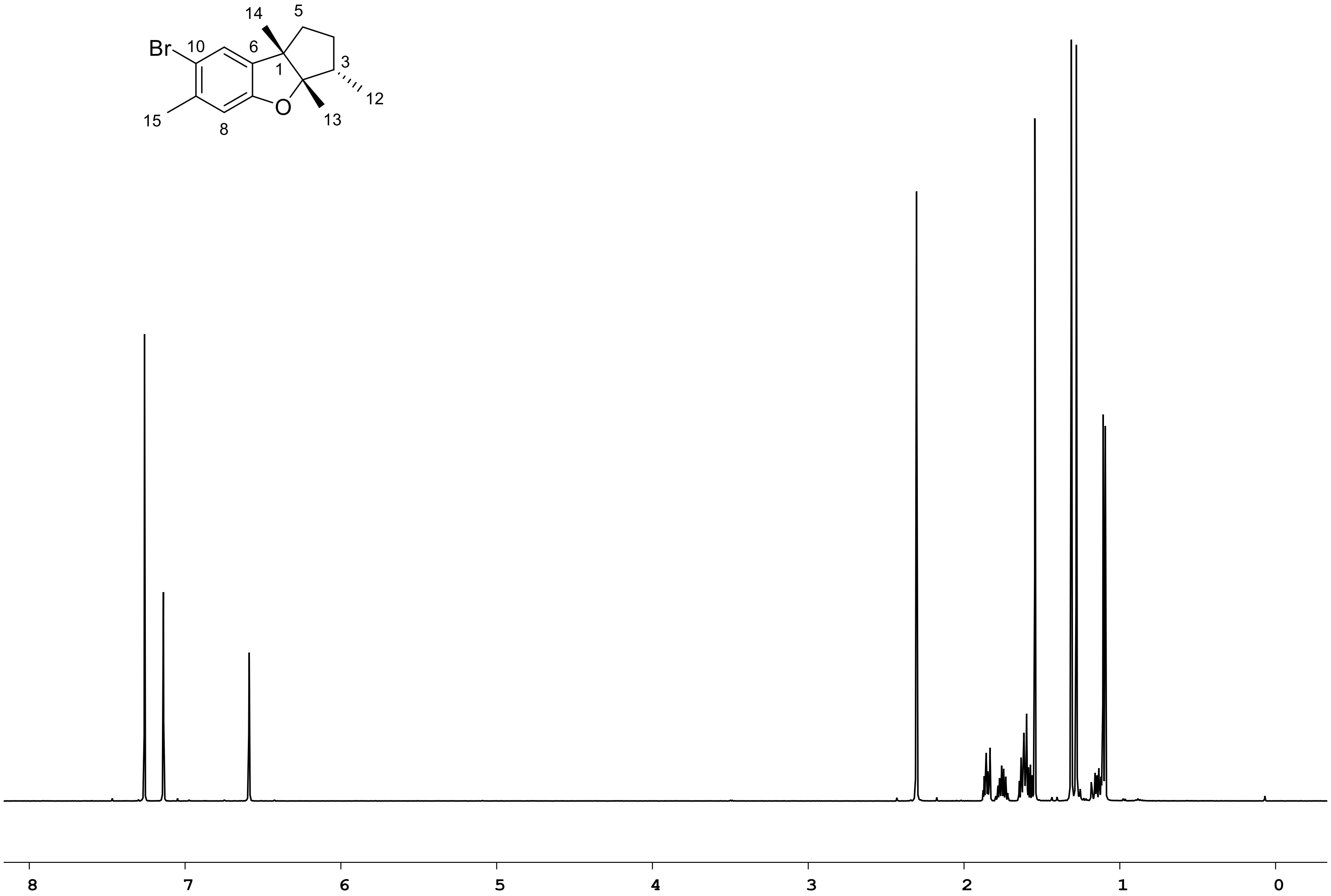

Figure S4. ^{1}H-NMR spectrum for α-bromocuparane (**4**) (500 MHz, $CDCl_3$).

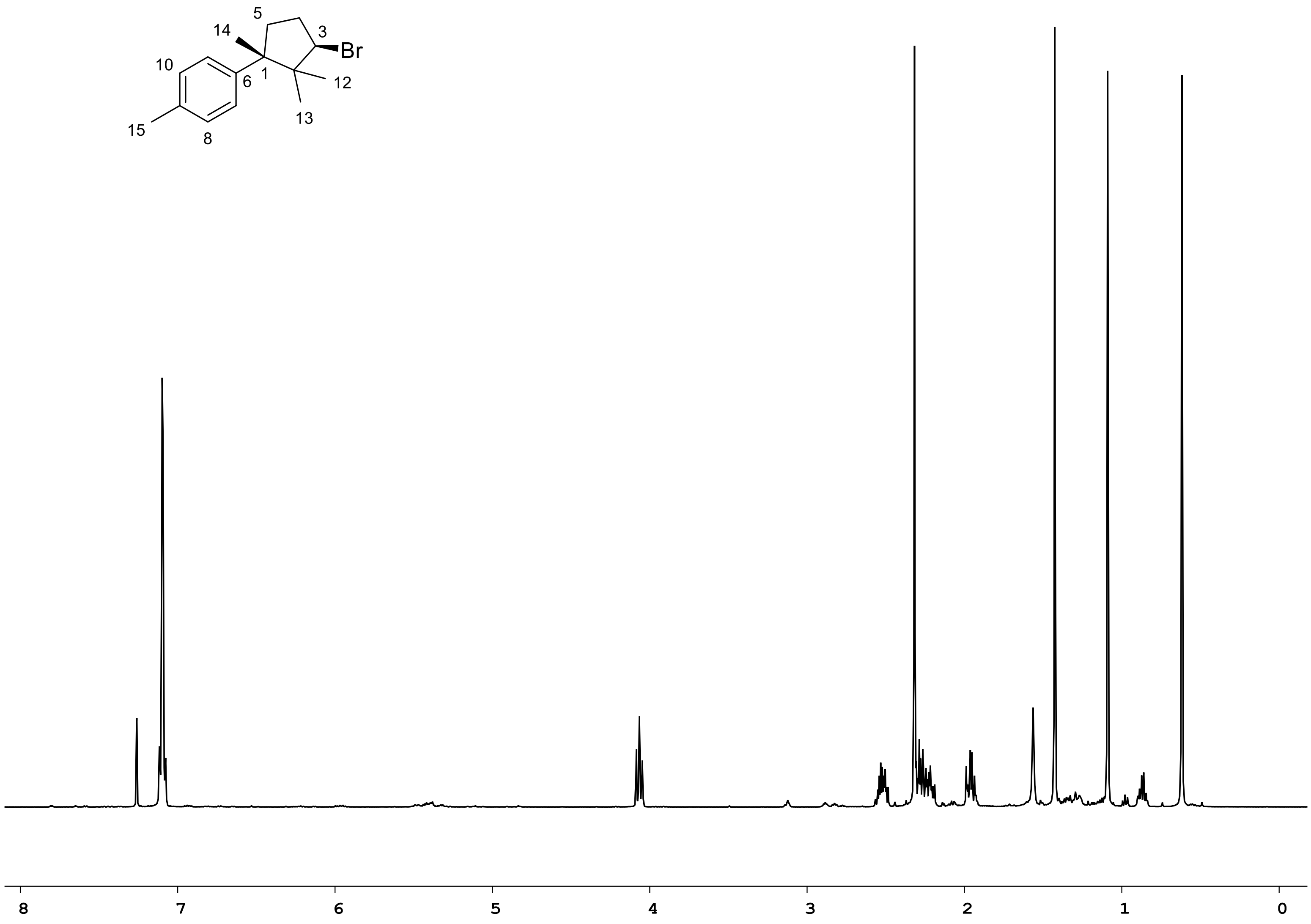

Figure S5. ^{1}H-NMR spectrum of α-isobromocuparane (**5**) (500 MHz, $CDCl_3$).

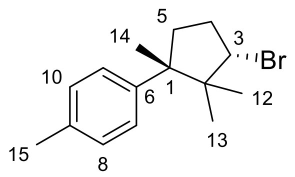

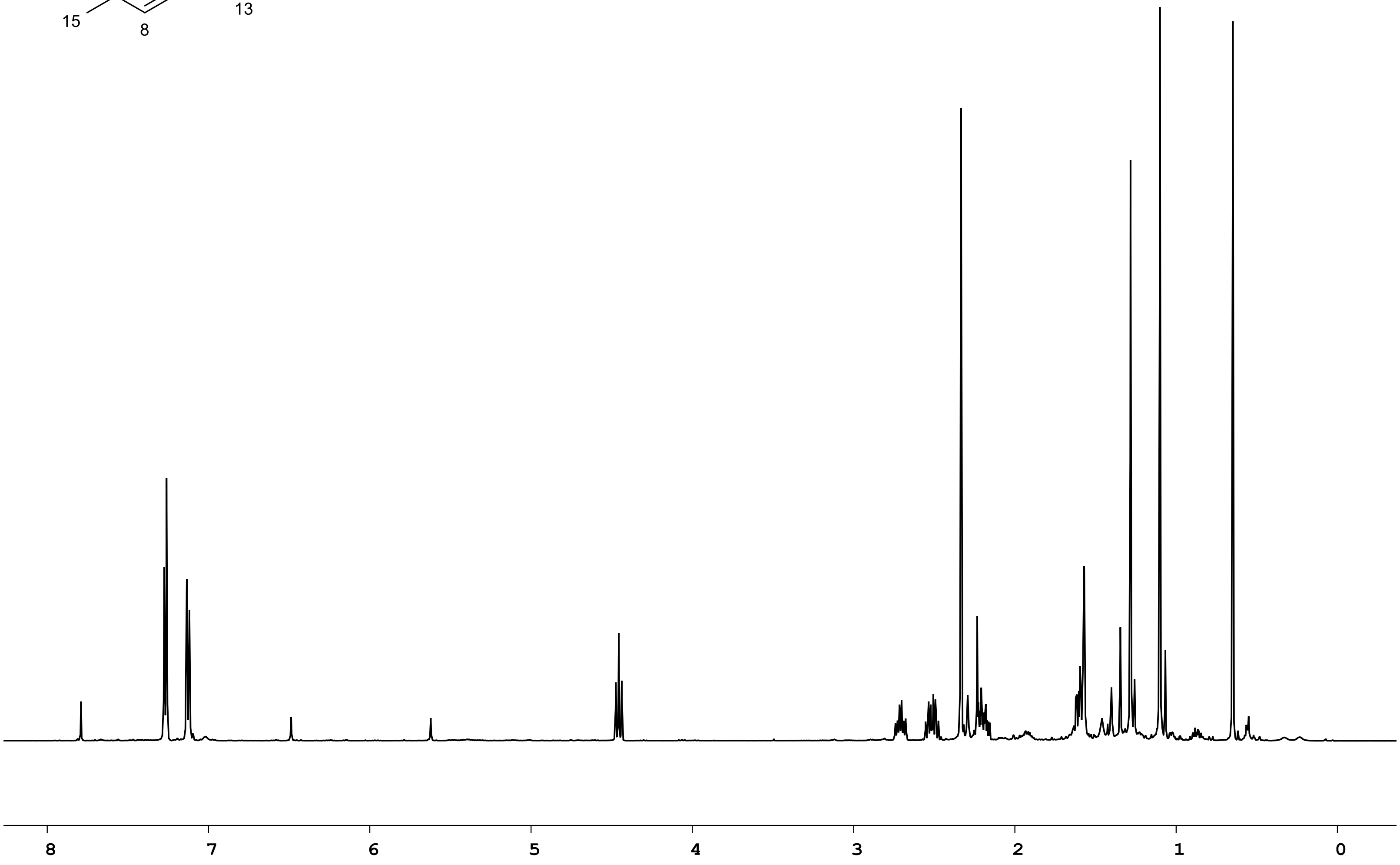

Figure S6. ^{1}H-NMR spectrum of 8-bromoaplysin (**6**) (600 MHz, $CDCl_3$).

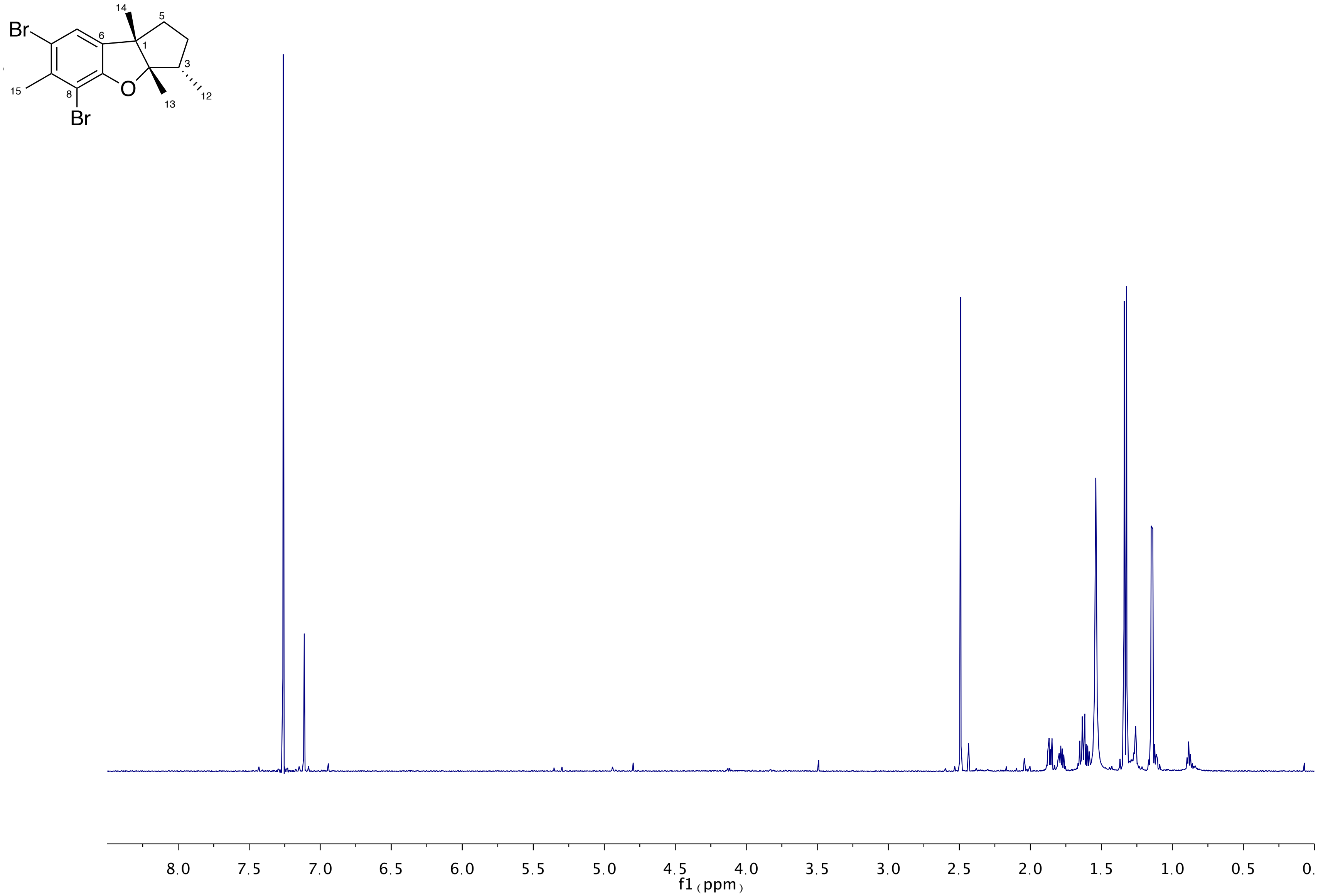

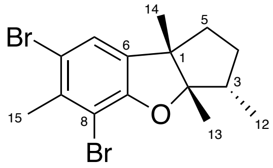

Figure S7. COSY spectrum of 8-bromoaplysin (**6**) (600 MHz, $CDCl_3$).

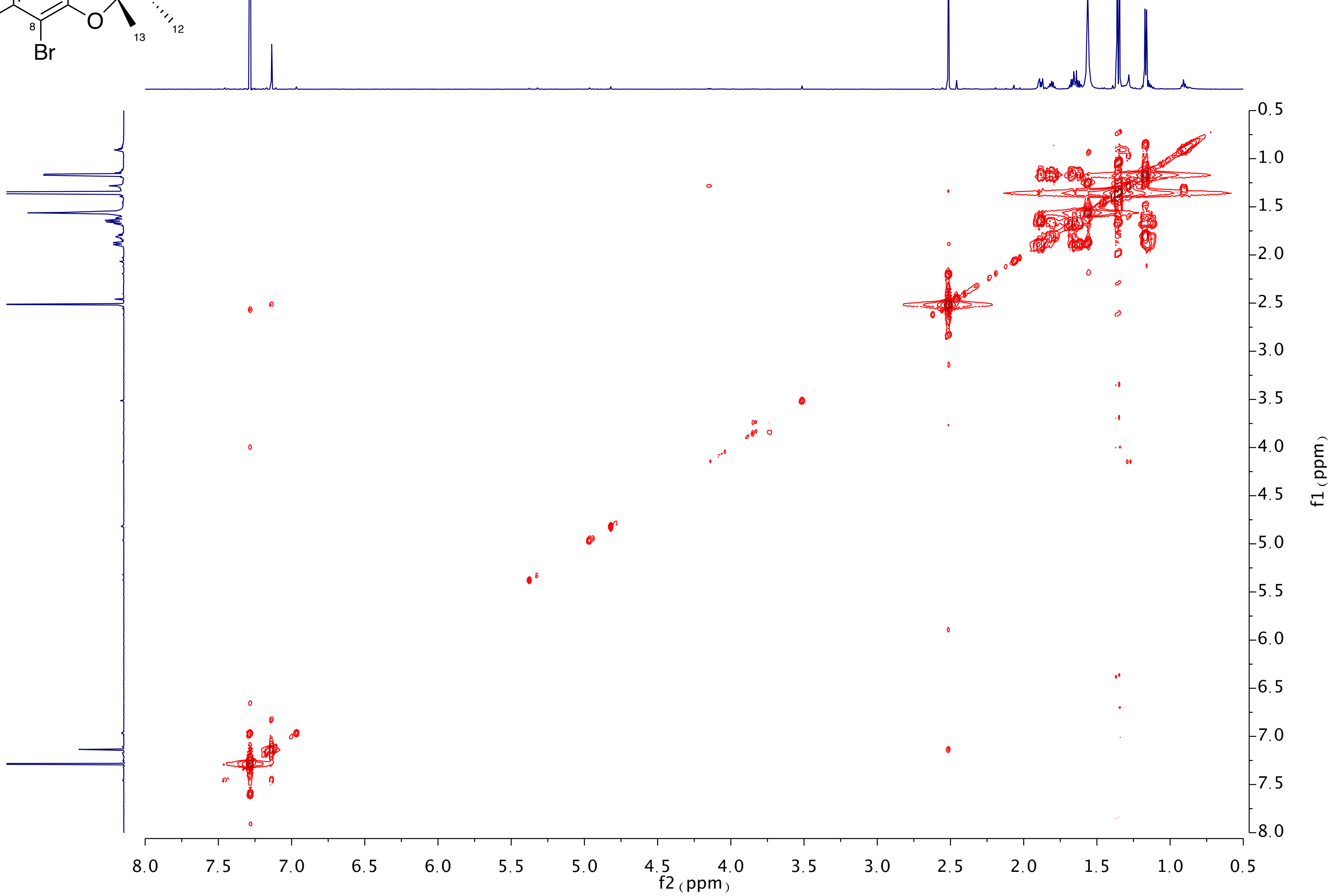

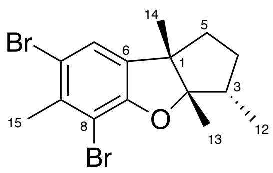

Figure S8. HSQC-ed spectrum of 8-bromoaplysin (**6**) (600 MHz, $CDCl_3$).

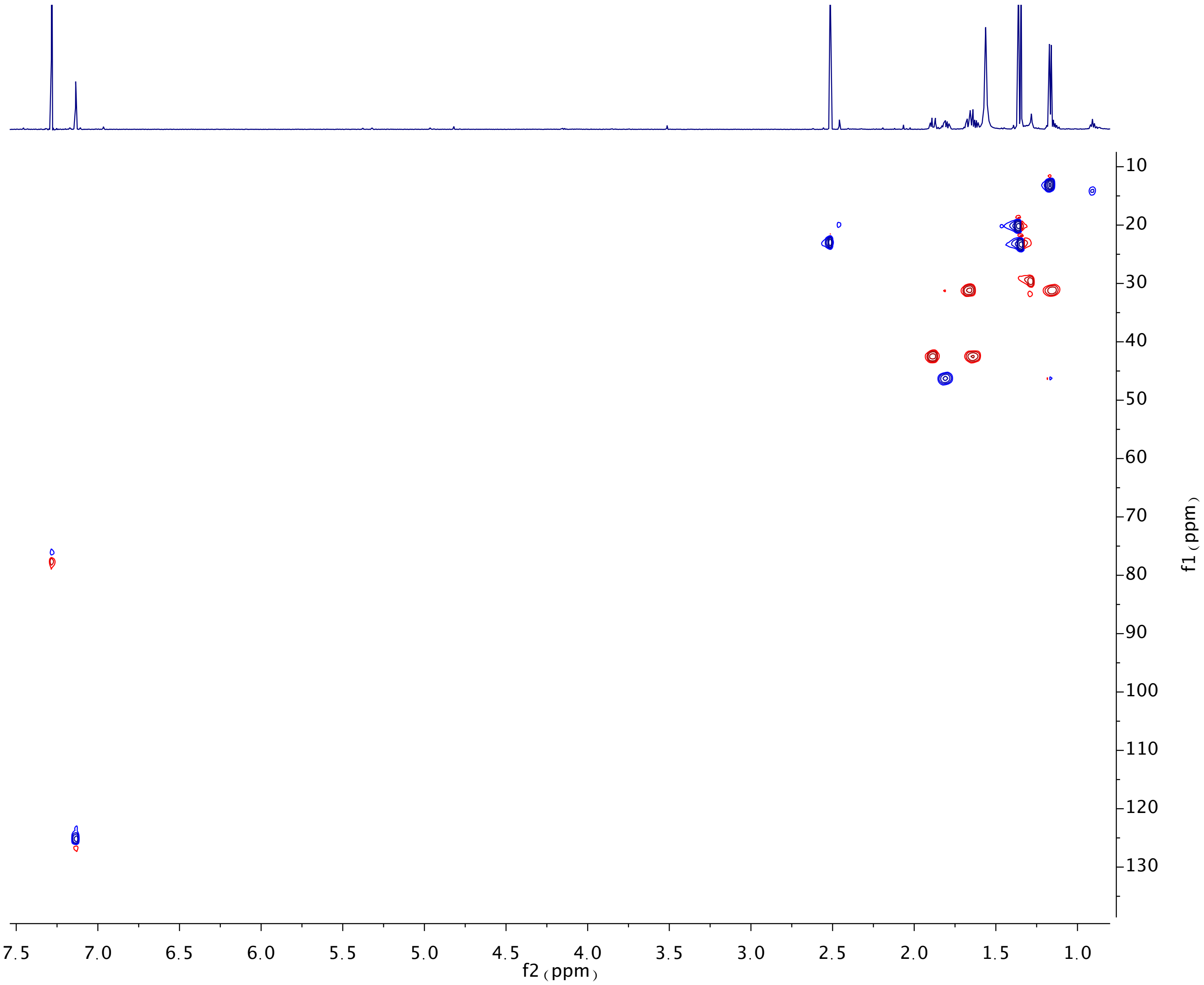

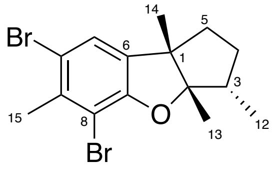

Figure S9. HMBC spectrum of 8-bromoaplysin (**6**) (600 MHz, $CDCl_3$).

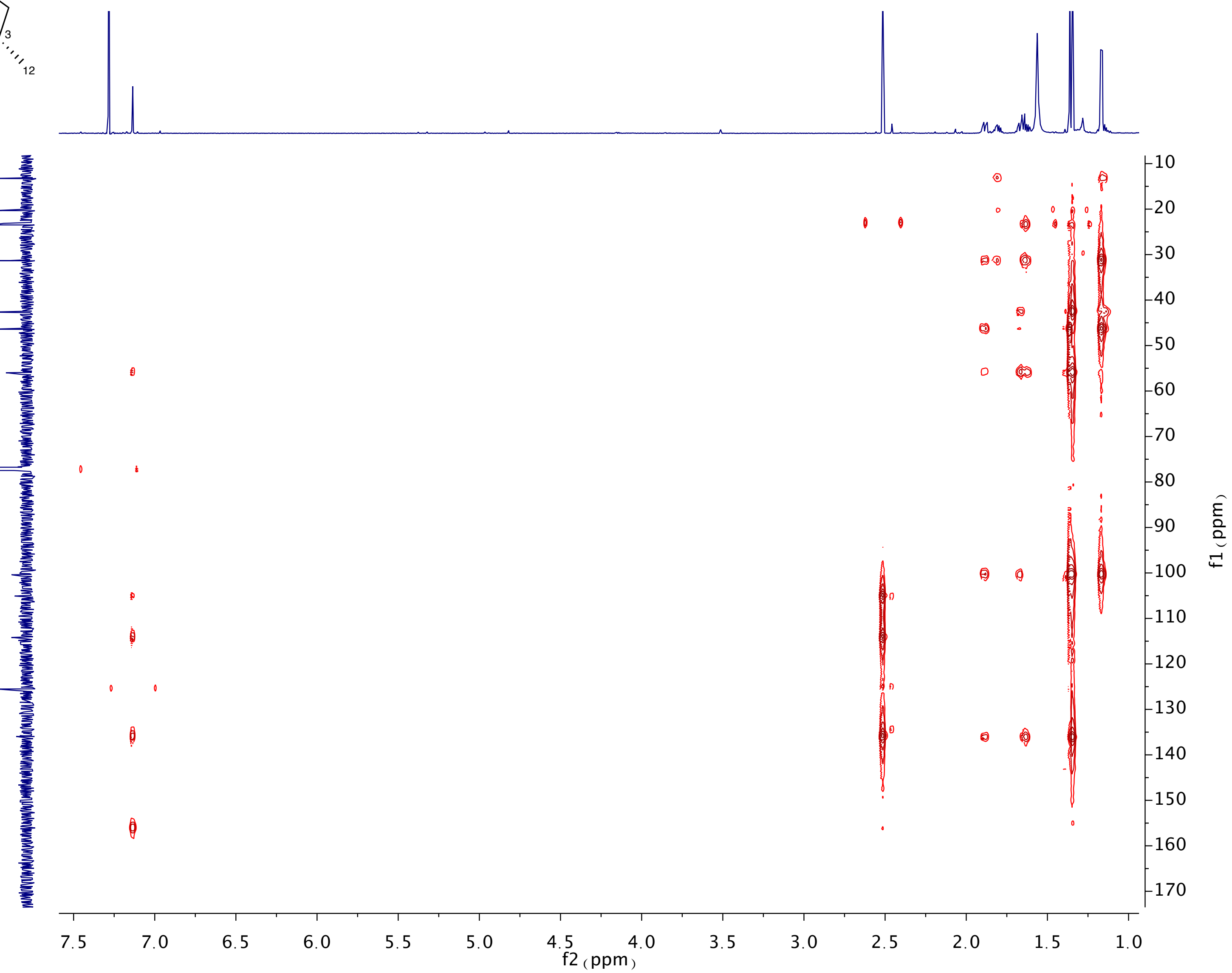

Figure S10. ^{13}C NMR spectrum of 8-bromoaplysin (**6**) (150 MHz, $CDCl_3$).

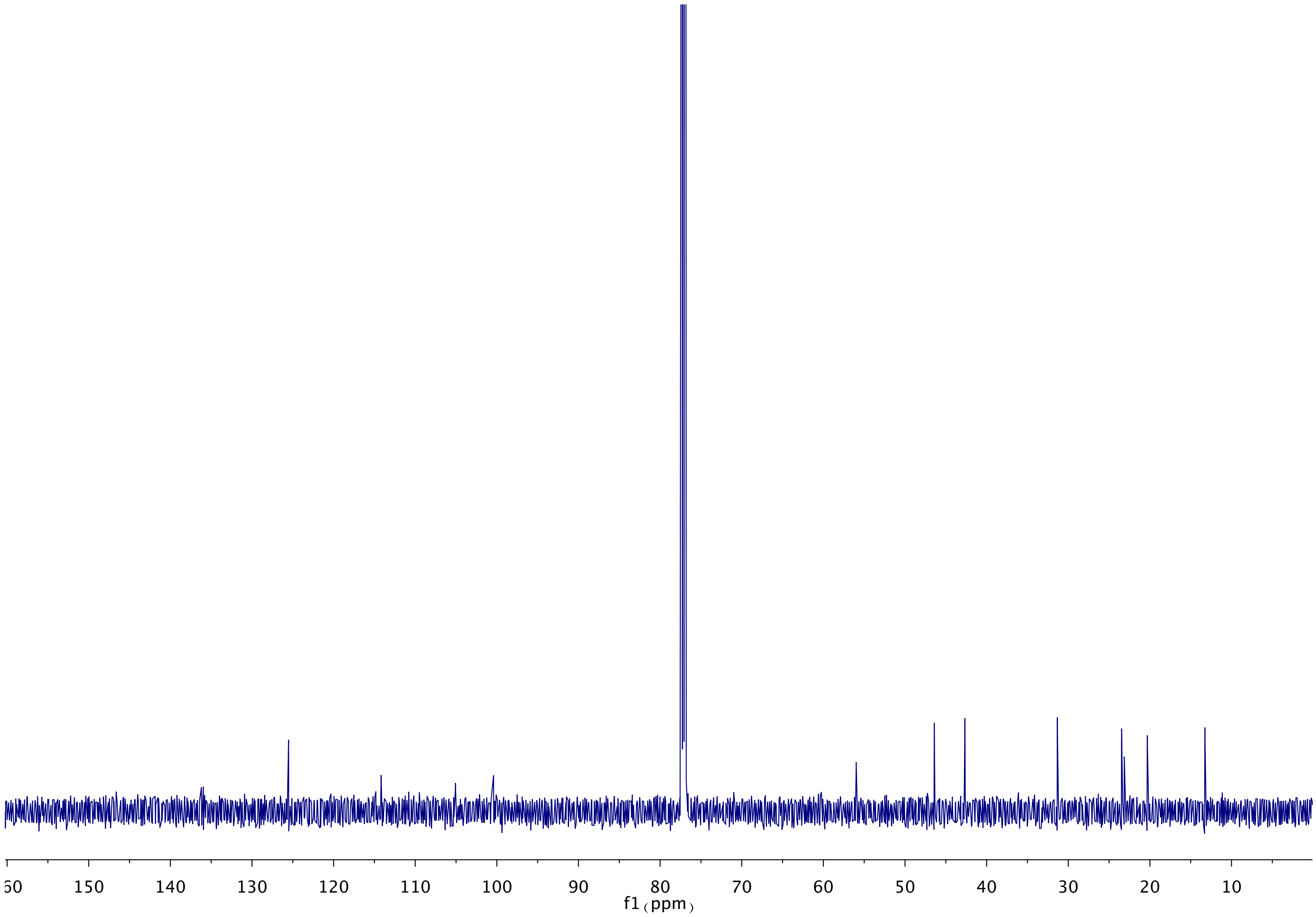

Figure S11. HREIMS spectrum of 8-bromoaplysin (**6**).

Elemental Composition Report

Page .

Multiple Mass Analysis: 1974 mass(es) processed - displaying only valid results
Tolerance = 10.0 PPM / DBE: min = -1.5, max = 50.0
Selected filters: None

Monoisotopic Mass, Odd and Even Electron Ions
24346 formula(e) evaluated with 21 results within limits (all results (up to 1000) for each mass)
Elements Used:
C: 15-15 H: 18-18 O: 0-1 79Br: 0-2 81Br: 0-2

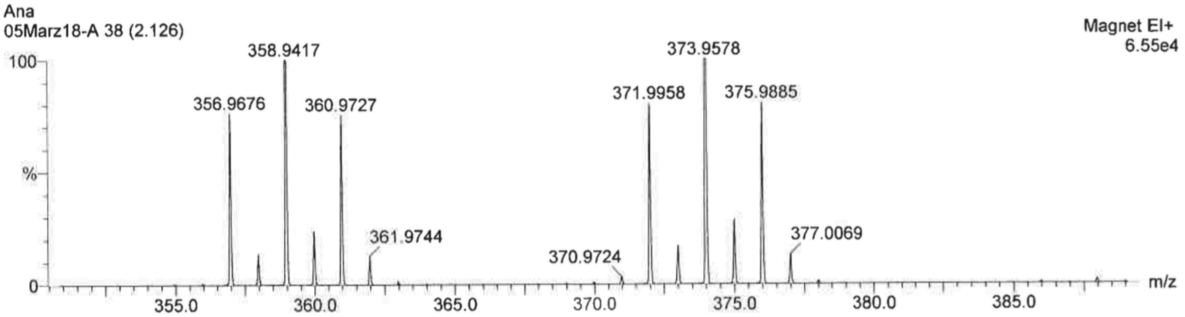

Minimum:	0.10				-1.5	
Maximum:	100.00		5.0	10.0	50.0	

Mass	RA	Calc. Mass	mDa	PPM	DBE	Formula
375.9718	58.99	375.9683	3.5	9.3	6.0	C15 H18 O 81Br2
375.9698	53.56	375.9683	1.5	4.0	6.0	C15 H18 O 81Br2
375.9677	47.91	375.9683	-0.6	-1.6	6.0	C15 H18 O 81Br2
375.9656	42.16	375.9683	-2.7	-7.2	6.0	C15 H18 O 81Br2
373.9723	100.00	373.9704	1.9	5.1	6.0	C15 H18 O 79Br 81Br
373.9702	100.00	373.9704	-0.2	-0.5	6.0	C15 H18 O 79Br 81Br
373.9682	100.00	373.9704	-2.2	-5.9	6.0	C15 H18 O 79Br 81Br
371.9752	57.29	371.9724	2.8	7.5	6.0	C15 H18 O 79Br2
371.9732	54.26	371.9724	0.8	2.2	6.0	C15 H18 O 79Br2
371.9711	49.60	371.9724	-1.3	-3.5	6.0	C15 H18 O 79Br2
371.9690	44.63	371.9724	-3.4	-9.1	6.0	C15 H18 O 79Br2
359.9757	22.43	359.9734	2.3	6.4	6.0	C15 H18 81Br2
359.9737	22.78	359.9734	0.3	0.8	6.0	C15 H18 81Br2
359.9716	23.04	359.9734	-1.8	-5.0	6.0	C15 H18 81Br2
357.9781	13.21	357.9755	2.6	7.3	6.0	C15 H18 79Br 81Br
357.9761	13.42	357.9755	0.6	1.7	6.0	C15 H18 79Br 81Br
357.9742	13.53	357.9755	-1.3	-3.6	6.0	C15 H18 79Br 81Br
357.9721	13.42	357.9755	-3.4	-9.5	6.0	C15 H18 79Br 81Br
355.9798	0.42	355.9775	2.3	6.5	6.0	C15 H18 79Br2
355.9778	0.43	355.9775	0.3	0.8	6.0	C15 H18 79Br2
355.9759	0.50	355.9775	-1.6	-4.5	6.0	C15 H18 79Br2

Figure S12. ^{1}H NMR spectrum of 3α-bromojohnstane (**7**) (600 MHz, $CDCl_3$).

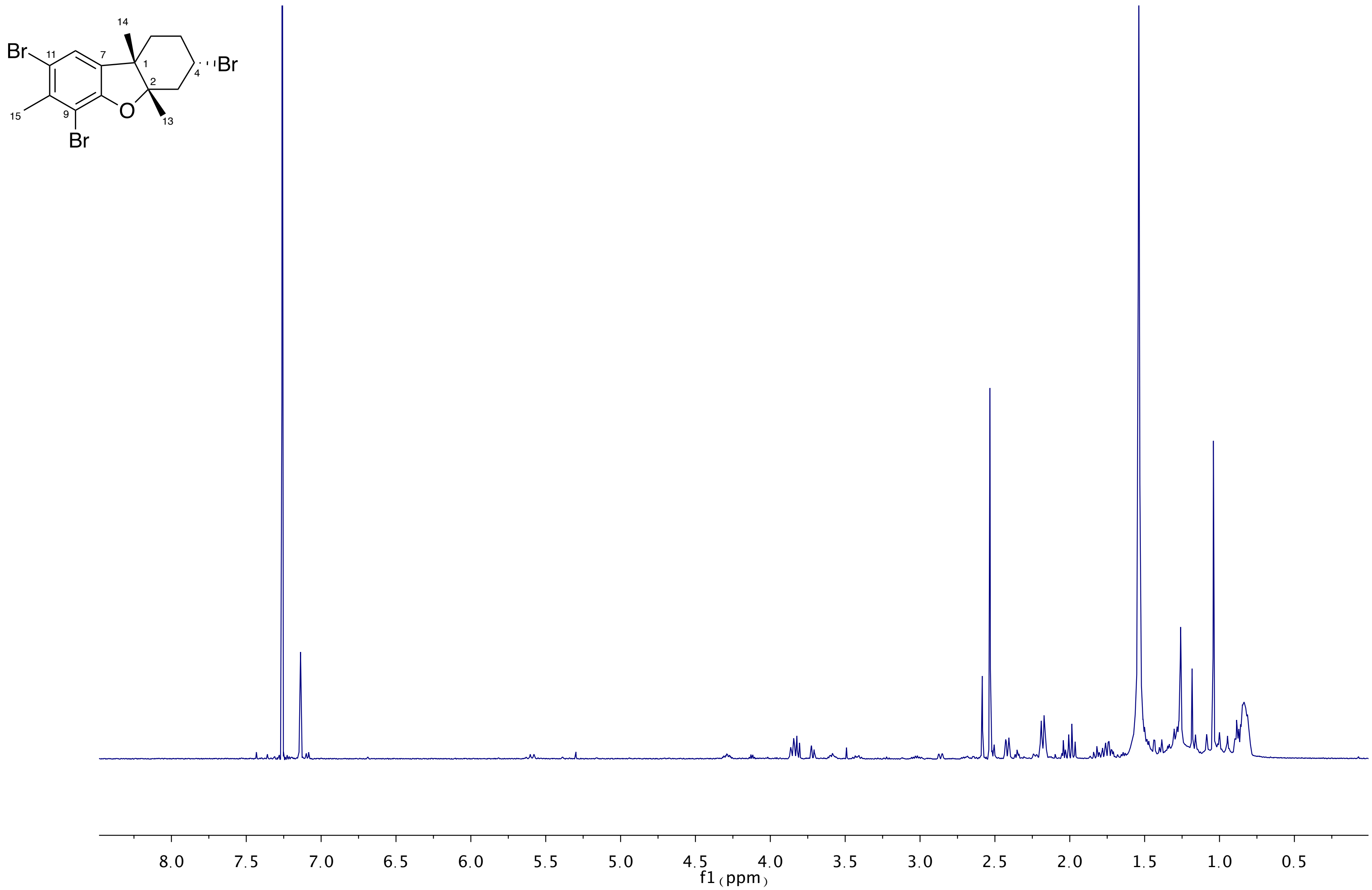

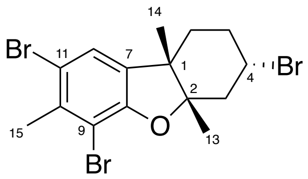

Figure S13. COSY spectrum of 3α–bromojohnstane (**7**) (600 MHz, $CDCl_3$).

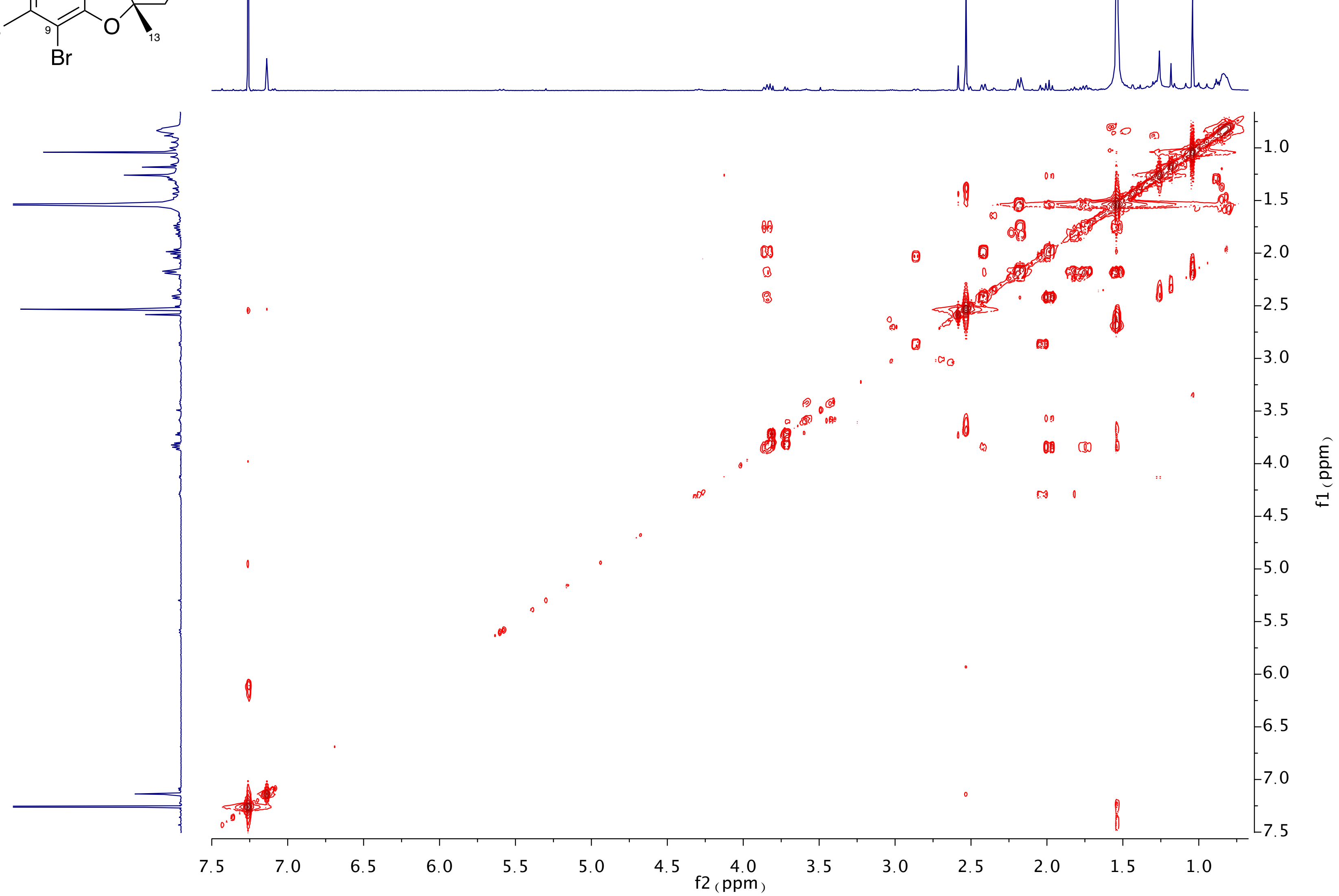

Figure S14. HSQC-ed spectrum of 3α-bromojohnstane (**7**) (600 MHz, $CDCl_3$).

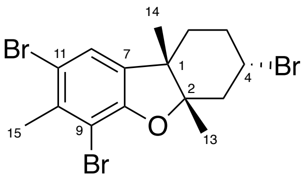

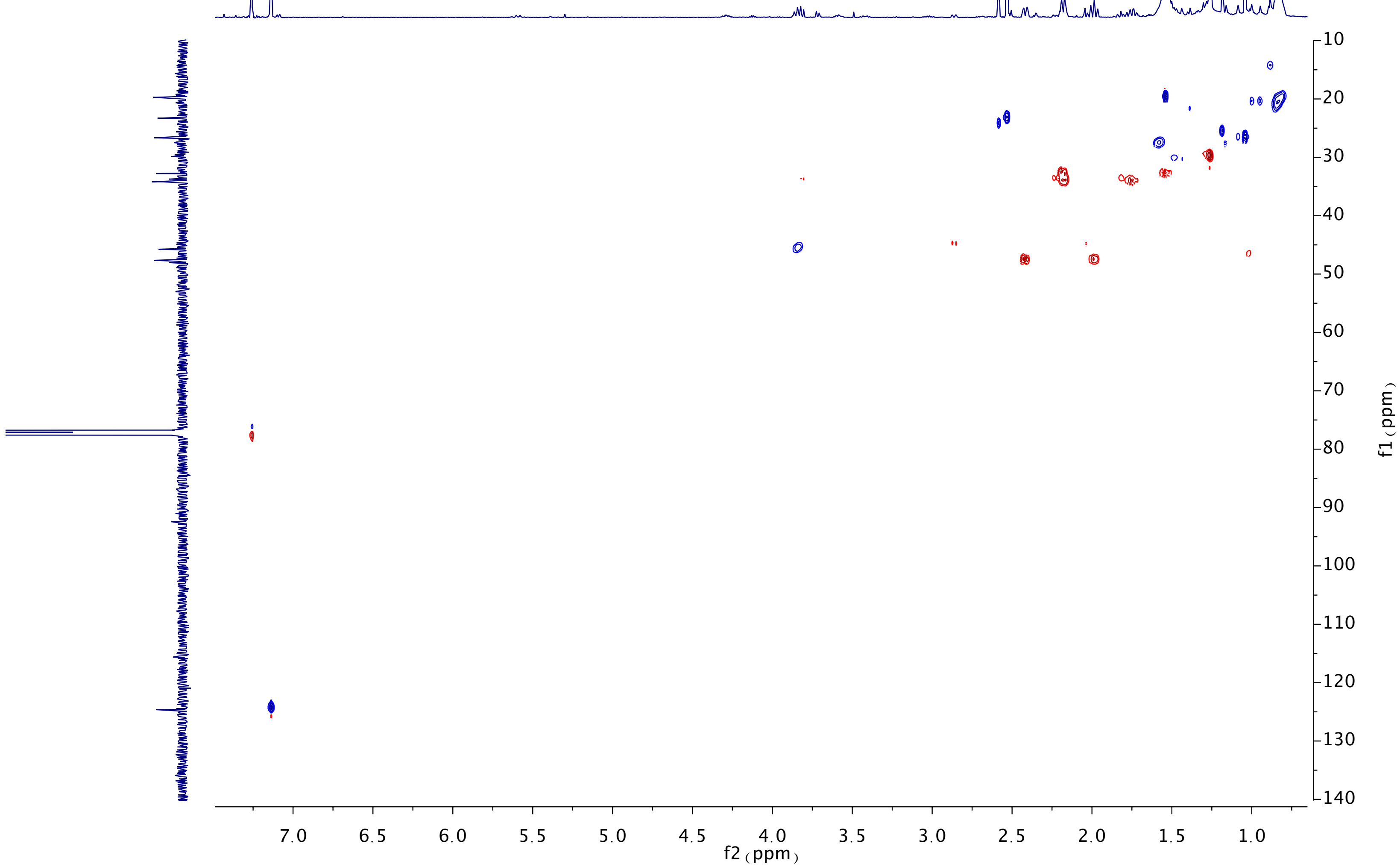

Figure S15. HMBC spectrum of 3α-bromojohnstane (**7**) (600 MHz, $CDCl_3$).

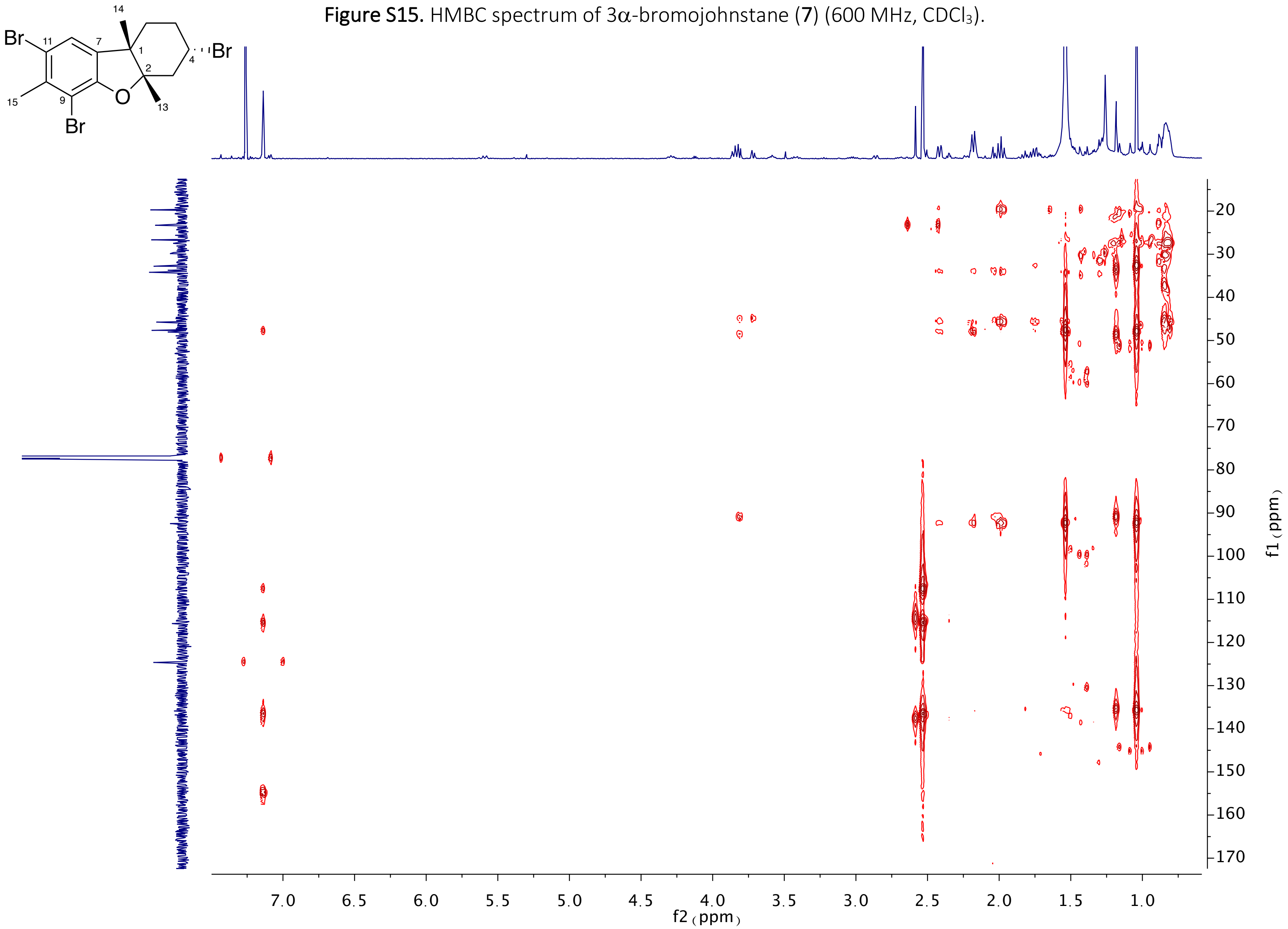

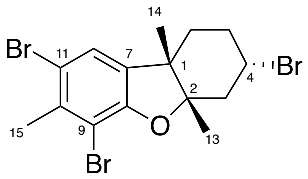

Figure S16. ^{13}C NMR spectrum of 3α-bromojohnstane (**7**) (150 MHz, $CDCl_3$).

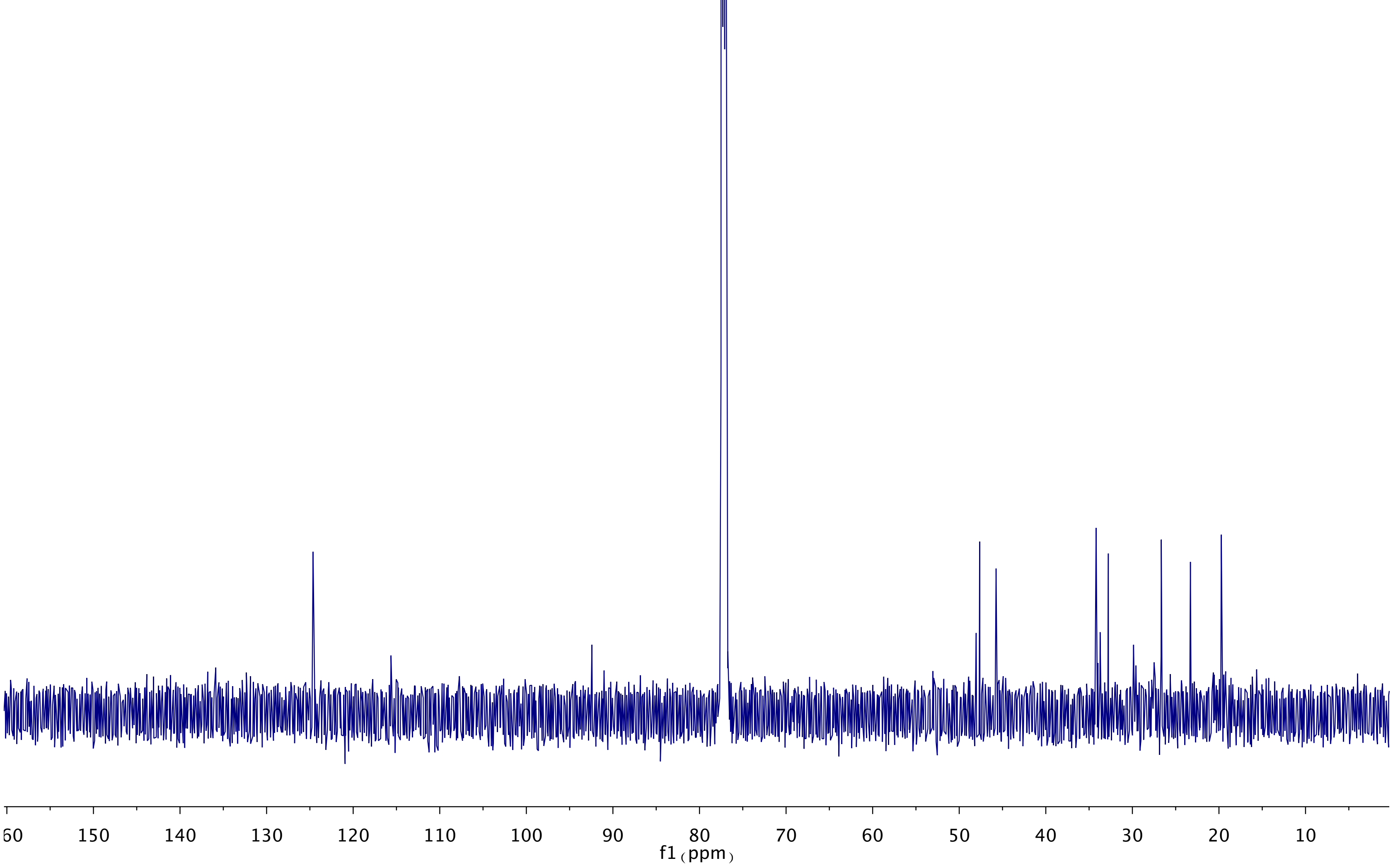

Figure S17. 1D-NOE experiments of 3α-bromojohnstane (**7**) (600 MHz, $CDCl_3$).

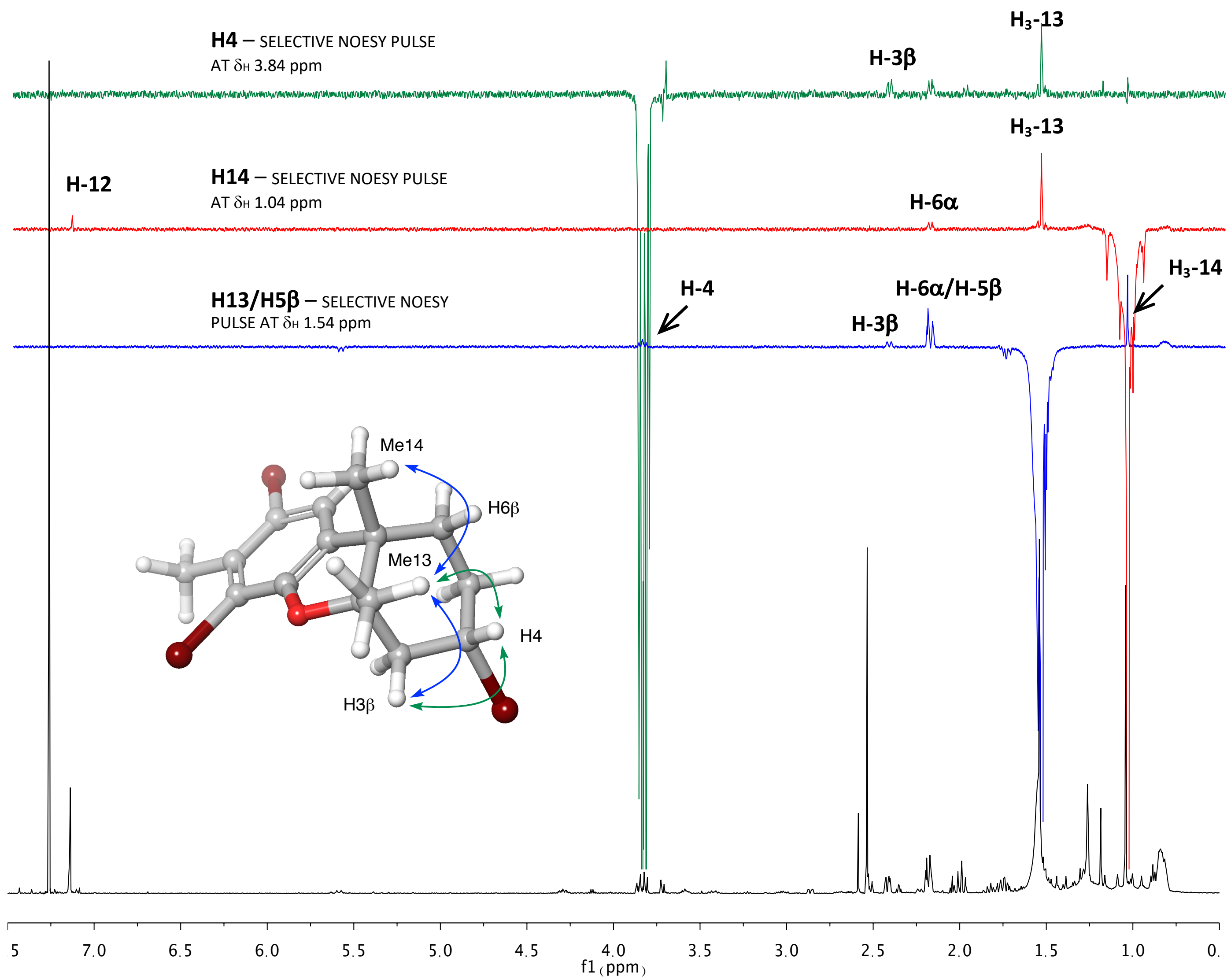

Figure S18. HREIMS spectrum of 3α-bromojohnstane (**7**).

Elemental Composition Report

Multiple Mass Analysis: 4297 mass(es) processed - displaying only valid results
Tolerance = 10.0 PPM / DBE: min = -1.5, max = 50.0
Selected filters: None

Monoisotopic Mass, Odd and Even Electron Ions
48456 formula(e) evaluated with 16 results within limits (all results (up to 1000) for each mass)
Elements Used:
C: 15-15 H: 17-17 O: 1-1 79Br: 0-3 81Br: 0-3

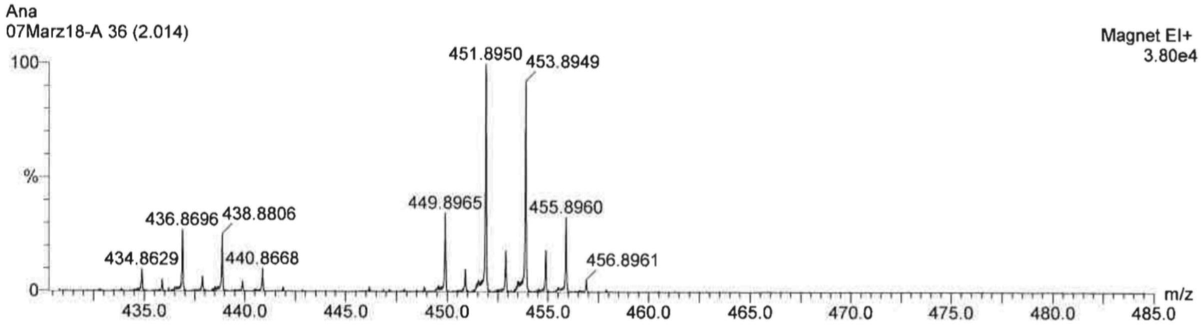

Minimum:	0.10				-1.5	
Maximum:	100.00		5.0	10.0	50.0	
Mass	RA	Calc. Mass	mDa	PPM	DBE	Formula
455.8809	24.09	455.8768	4.1	9.0	6.0	C15 H17 O 81Br3
455.8784	24.88	455.8768	1.6	3.5	6.0	C15 H17 O 81Br3
455.8758	25.80	455.8768	-1.0	-2.2	6.0	C15 H17 O 81Br3
455.8733	24.42	455.8768	-3.5	-7.7	6.0	C15 H17 O 81Br3
453.8823	74.42	453.8789	3.4	7.5	6.0	C15 H17 O 79Br 81Br2
453.8798	67.41	453.8789	0.9	2.0	6.0	C15 H17 O 79Br 81Br2
453.8773	61.33	453.8789	-1.6	-3.5	6.0	C15 H17 O 79Br 81Br2
453.8748	55.32	453.8789	-4.1	-9.0	6.0	C15 H17 O 79Br 81Br2
451.8850	80.74	451.8809	4.1	9.1	6.0	C15 H17 O 79Br2 81Br
451.8824	80.48	451.8809	1.5	3.3	6.0	C15 H17 O 79Br2 81Br
451.8800	78.16	451.8809	-0.9	-2.0	6.0	C15 H17 O 79Br2 81Br
451.8775	72.34	451.8809	-3.4	-7.5	6.0	C15 H17 O 79Br2 81Br
449.8865	28.64	449.8829	3.6	8.0	6.0	C15 H17 O 79Br3
449.8840	26.22	449.8829	1.1	2.4	6.0	C15 H17 O 79Br3
449.8816	23.43	449.8829	-1.3	-2.9	6.0	C15 H17 O 79Br3
449.8791	21.91	449.8829	-3.8	-8.4	6.0	C15 H17 O 79Br3

Figure S19. ^{1}H NMR spectrum of 8,10-dibromoisoaplysin (**8**) (500 MHz, $CDCl_3$).

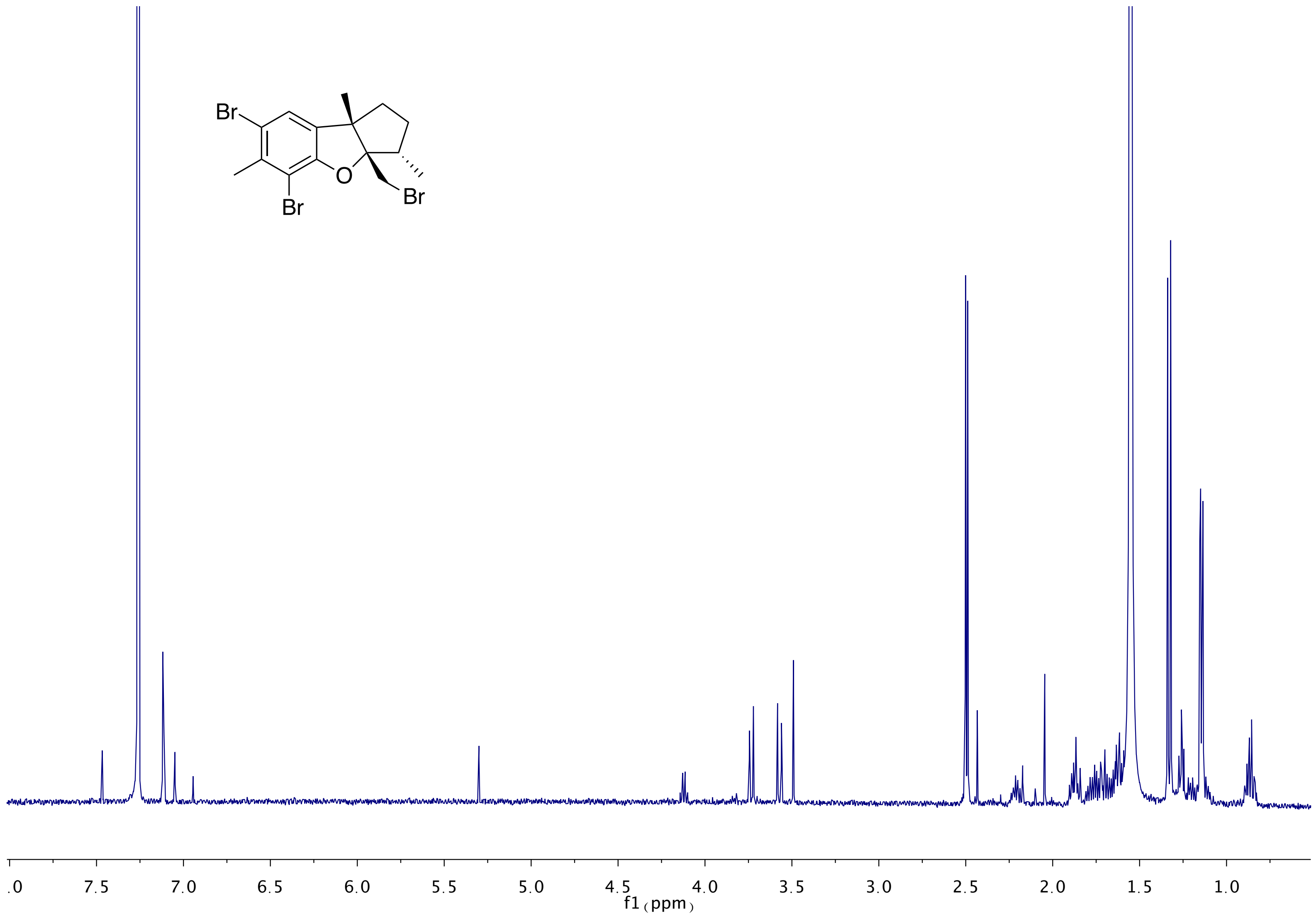

Figure S20. ^{1}H NMR spectrum of 8,10-dibromoaplysinol (**9**) (500 MHz, $CDCl_3$).

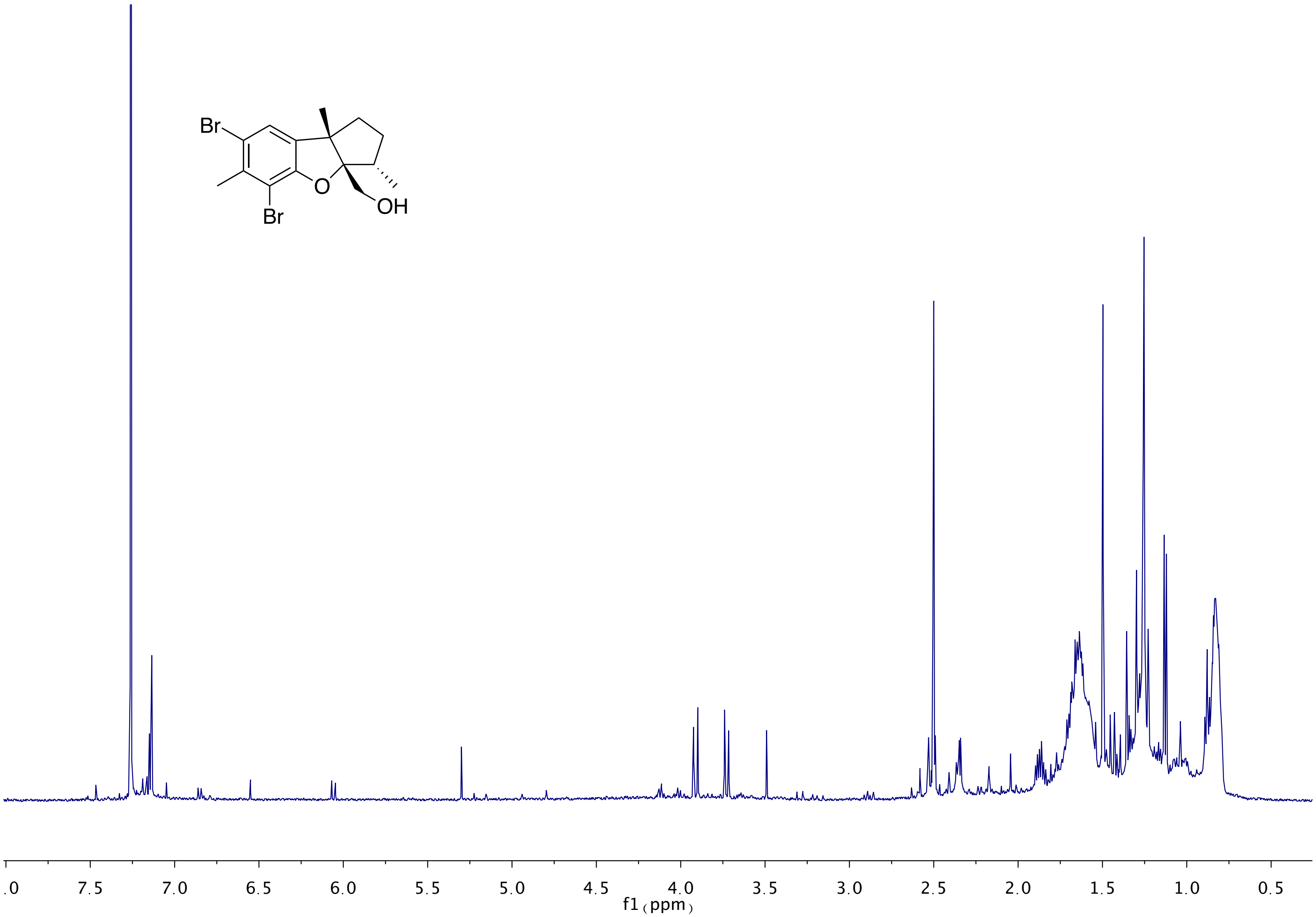

Supplement: Supplementary file 1 [file marinedrugs-16-00443-s001.pdf]
